# Neuromodulatory Effect of Sensorimotor Network Functional Connectivity of Temporal Three-Needle Therapy for Ischemic Stroke Patients with Motor Dysfunction: Study Protocol for a Randomized, Patient-Assessor Blind, Controlled, Neuroimaging Trial

**DOI:** 10.1155/2021/8820324

**Published:** 2021-01-04

**Authors:** Ning Zhao, Hong Zhang, Tongyan Liu, Jiao Liu, Yun Xiang, Guojian Shu, Chunzhen Li, Jingwen Xie, Lidian Chen

**Affiliations:** ^1^Fujian University of Traditional Chinese Medicine, Fuzhou 350122, China; ^2^The 6th Affiliated Hospital of Shenzhen University Health Science Center, Shenzhen Nanshan District People's Hospital, Shenzhen 518052, China; ^3^Hunan University of Traditional Chinese Medicine, Changsha 410007, China

## Abstract

**Background:**

The clinical efficacy of temporal three-needle therapy for stroke dysfunction has been previously demonstrated in China. However, the central mechanism of temporal three-needle therapy remains unclear. Temporal three-needle projects the sensory cortex and the motor cortex, which may impact the cortex function. Current studies seldom focus on it. Hence, according to the “scalp-cortex corresponding theory,” the underlying mechanism of temporal three-needle remains a domain for further research.

**Methods:**

This trial is designed to provide objective and visual evidence for the neuromodulatory effect and neuroimaging mechanism of temporal three-needle therapy for stroke patients. This ongoing study is a prospective, randomized, controlled, patient-assessor blind, single-center, neuroimaging trial involving two-parallel patient groups and a healthy control group. Forty eligible patients will be recruited from Shenzhen Nanshan District People's Hospital and randomized into either the experimental group or the control group. Twenty healthy volunteers will be recruited in the healthy control group and undergo baseline magnetic resonance imaging scans without any intervention. Patients in the control group will receive acupuncture at Dingnieqianxiexian (MS6), in addition to basic medicine and rehabilitative treatments. Patients in the experimental group will receive temporal three-needle therapy plus basic medicine and rehabilitative treatments 5 days per week, 10 sessions over two consecutive weeks. The primary outcome is resting-state functional connectivity, and the secondary outcomes are regional homogeneity, amplitude of low-frequency fluctuations, Fugl–Meyer assessment of the upper limb, and modified Barthel Index. All outcome measures will be assessed at baseline and after 2 weeks of intervention. *Discussion*. The results will explore the neuromodulatory effects and illustrate the central mechanism of temporal three-needle treatment from the network-level viewpoint of sensorimotor network functional plasticity and promote widespread application in real-world practice. This trial was registered at Chinese Clinical Trial Registry on 14 March 2018 with ChiCTR1800015209.

## 1. Introduction

Stroke is the second leading cause of death, and Kim et al reported that stroke remained the third-leading cause of years-of-potential-life lost worldwide [1]. The stroke incidence ranged from 76 per 100,000 population per year up to 119 per 100,000 population per year in different countries [2], and it is expected that the incidence of stroke will continue to increase as the population ages [3]. There are about two million new patients of stroke annually in China [4]. Most of the stroke survivors are associated with functional disabilities in China [5], and motor dysfunction is the most significant symptom, which results in different levels of dysfunction (including muscle atrophy, joint stiffness, shoulder pain, deep vein thrombosis of the lower limb, long-term brakes, infection, anxiety, and depression) and disability in daily life. The stroke impact in China is more severe compared to the worldwide average levels. DALYs (disability adjusted life-years) caused by stroke rank 3rd in global epidemiologic study and first in China [6]. Overall poststroke care costs were the highest in the USA ($4850/per patient month) [7], which indicates that stroke not only brings pain to patients themselves but also imposes a great financial burden on families and communities in most countries, which has become a critical public health problem.

In 2002, the World Health Organization recommended acupuncture as a therapy for stroke [8]. Temporal three-needle acupuncture is one of Jin's three-needle therapy systems created by Professor Jinrui in China. The temporal I needle is located on the hairline 2 cun above the apex of the ear; the temporal II needle and the temporal III needle are located 1 cun from the temporal I needle on either side in the horizontal direction [9]. Temporal three-needle therapy integrates the meridian acupoint theory in Chinese traditional medicine, modern neurological theory, and anatomical rationale [10, 11]. The location of the temporal three-needle is highly overlapped with the temporal bone seams, which is a crevice left by the congenital development process [12]. Additionally, the nerve is extended into the skull through the gap of the skull, which can effectively mobilize the neuron activity after acupuncture, and the position of the temporal three-needle ranged including the precentral gyrus and postcentral gyrus could widely affect the motor and sensory areas [13]. Meanwhile, the bioelectric effect of acupuncture at the location can be transmitted to the cerebral cortex, which can quickly rebuild the function of injured neurons in the functional area and strengthen the compensatory ability of the brain [11]. Moreover, the temporal bone is much thinner than other skulls, and its suture is mostly densest; acupuncture sensation is easier to be conducted, and the acupuncture effect is better. As a result, the acupoint selection of temporal three-needle has a certain representative. In real-world clinical practice, acupuncture on the left temporal three-needle treats right limb paralysis and acupuncture on the right temporal three-needle for the left paralysis, which is widely used in the poststroke rehabilitation in China [11, 14–18], especially motor dysfunction [14, 15, 19].

Because the study design is a superiority trial and stroke patients conventionally receive acupuncture at the scalp acupoint in current clinical practice in China, placebo (sham) stimulation or a blank control may violate the patient willingness. This trial will choose Dingnieqianxiexian (MS6) in the International Standardization Scheme for Scalp Acupuncture Points as the positive comparator in the control group. MS6 is an international standardized scalp acupoint, which refers to a diagonal line from Qianshencong (EX-HN1) to Xuanli (GB6) [20], and is projected to the precentral gyrus [21]; it is responsible for the autonomous movement of the limbs [20]. Studies have indicated that, on the basis of body acupuncture, temporal three-needle therapy is superior to acupuncture at MS6 with respect to daily life activities [22], motor function [22], and motor-related sensory disturbances [19].

However, the central mechanism of temporal three-needle therapy on stroke recovery remains unclear, especially in neuroimaging. From the last several years, with the rapid development of medical imaging technology, magnetic resonance imaging (MRI) has gradually become one of the main methods for studying the mechanism of acupuncture. Resting-state fMRI (rs-fMRI) only requires the subjects to stay still and close their eyes while scanning and keep their body as immobile as possible without thinking about anything. Rs-fMRI reflects intrinsic functional patterns and the spontaneous activity of the brain's cortex [23], which is a useful technique for studying the mechanism of acupuncture [24]. In this trial, we plan to apply rs-fMRI to explore the central mechanism of temporal three-needle treatment.

Previous neuroimaging studies demonstrated that motor disorders in stroke patients were associated with functional connectivity (FC) abnormalities in the sensorimotor network or sensorimotor cortex [25, 26]. Moreover, functional recovery after stroke is associated with preserved FC of motor to nonmotor networks [27]. Acupuncture can increase the default mode network and sensorimotor network functional connectivity with pain-, affective-, and memory-related brain areas compared with sham acupuncture [24]. Several studies have also shown that acupuncture increases the sensorimotor network connectivity with pain-related brain regions in healthy adults or inhibits neuroinflammation in the sensorimotor cortex after ischemic stroke [28, 29]. However, the neuroimaging mechanism based on temporal three-needle therapy in hemiplegic patients with stroke remains unclear. The theoretical basis of scalp acupuncture is mainly based on the two aspects [20]: (1) the traditional Zangfu meridians theory and (2) scalp-cortex corresponding theory. According to the “scalp-cortex corresponding theory,” the temporal three-needle projects the cortex of the sensory area and the motor area on the temporal ear, and the bioelectric effect produced by acupuncture in this area is transmitted to the cerebral cortex, which can impact the function of the cerebral cortex [11]. Given the abovementioned viewpoints, we hypothesize that the underlying neuroimaging mechanism on neuromodulatory effects in temporal three-needle acupuncture may be related to sensorimotor network functional connectivity.

Therefore, our first objective is to assess the neuromodulatory effect of temporal three-needle therapy on resting-state regional brain activity, whole-brain activity, and motor function. Our second objective is to explore the relationship between alterations in functional connectivity at the sensorimotor network level and changes in behavioral performances to demonstrate the underlying neuroimaging mechanism of temporal three-needle treatment.

## 2. Methods

### 2.1. Study Design and Setting

This study will be a two-week, prospective, randomized, patient-assessor blind, controlled, single-center, superiority, neuroimaging trial with two-parallel patient groups and a healthy control group. This study will take place mainly in Shenzhen Nanshan District People's Hospital, Shenzhen, Guangdong Province, China. In this trial, we will recruit subjects into two categories. The first category will comprise healthy controls, and the second will comprise patient subjects. A total of 20 age-, gender-, and education-matched healthy subjects will be recruited into the healthy control group. The purpose of healthy control recruitment is to establish a baseline normal MRI data set to facilitate comparison with the MRI data of the patients' groups. The healthy control group will receive baseline MRI scans without any intervention. Forty eligible patients will be randomized into two groups: (1) the experimental group and (2) the control group, with a 1 : 1 allocation ratio. All patients will take medicine for secondary prevention of stroke (e.g., antihypertensive, hypoglycemic, antiplatelet, and hypolipidemic drugs) and conventional rehabilitative treatments according to the Chinese stroke rehabilitation treatment guidelines [5] (e.g., physical therapy (PT) and occupational therapy (OT)); these treatments will be consistent across the patient groups. In the control group, patients will receive acupuncture at MS6 following basic drug treatments and conventional rehabilitative treatments. Patients in the experimental group will receive temporal three-needle acupuncture 5 days per week, 10 sessions over 2 consecutive weeks, plus basic drug treatments and conventional rehabilitative treatments. Acupuncturists with at least 10 years of clinical experience will perform acupuncture therapy (including temporal three-needle therapy and MS6 therapy). Professional rehabilitative therapists with above 5 years of clinical experience will conduct conventional rehabilitative treatments (PT and OT) for the patients.

**Figure 1:**
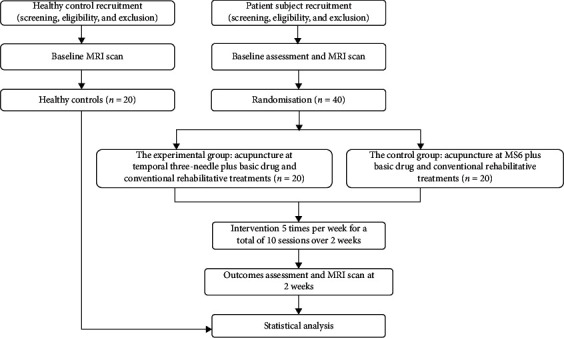
Planned flowchart of the trial.

The planned flowchart of the trial is shown in Figure 1. The timeline for assessment is provided in Table 1. This study protocol is in compliance with the requirements of the Helsinki Declaration. Ethical approval was provided by the Ethics Committee of Shenzhen Nanshan District People's Hospital in February 2018. In addition, Shenzhen Nanshan District People's Hospital is also carrying out this trial and is responsible for the coordination of all departments' activities (e.g., study protocol registry, staff training, informed consent implementation, and data management). This protocol follows Standard Protocol Item: Recommendations for Interventional Trials (SPIRIT) 2013 [30] (see Additional File 1 in Supplementary Materials available here) and the Consolidated Standards of Reporting Trials (CONSORT) 2010 statement for nonpharmacological interventions [31].

### 2.2. Sample Size Calculation

Our primary outcome will be resting-state FC measured by rs-fMRI. Because millions of voxels are used to indirectly estimate the blood oxygen level-dependent (BOLD) signal and traditional power calculations are of no significance [32], we calculated the sample size according to the relevant published articles. Previous rs-fMRI study of the acupuncture mechanisms in stroke had a sample size of 20 cases for the healthy control group [33]. Moreover, a study on the effects of sample size on cerebral response to acupuncture with fMRI had shown that the imaging results in 17 healthy controls were similar to the results of studies with 21 cases [34], which may suggest that approximately 20 subjects maybe a relatively common choice in rs-fMRI studies after acupuncture. The sample size in acupuncture mechanism study using rs-fMRI with stroke patients typically ranges from a few cases to approximately 20 cases [33, 35–37]. Because this trial is a neuroimaging study, the primary outcome will be based on the subjects' MRI image processing, and patients with left cerebral ischemia accompanied by right limb hemiplegia will be enrolled in this study so as to ensure the relative consistency of cerebral ischemia lesions and reveal the neuroimaging mechanism more regularly.

Under the condition of left brain ischemia stroke with right limb hemiplegia in this study, we plan to enroll 20 patients diagnosed with ischemia stroke in each patient group (20 in the experimental group and 20 in the control group) and 20 healthy volunteers in the healthy control group.

### 2.3. Participants and Recruitment

Forty patients diagnosed with stroke will be recruited from Shenzhen Nanshan District People's Hospital. Three trial staff will conduct the recruitment in accordance with the inclusion and exclusion criteria to determine whether the patients are eligible for the study. Patients will be recruited through oral recruitment during daily consultation, advertisements, WeChat in Shenzhen, and volunteer recommendations. All patients' subjects will undergo a baseline evaluation, including complete the collection of demographic and clinical data.

After completion of the recruitment of the forty patients, a total of 20 age-, gender-, and education-matched healthy subjects will be recruited from the community-dwelling population in the Nanshan District in Shenzhen, Guangdong Province, China, according to the demographic characteristics of the experimental group. The recruitment methods will be the same as that of the patients.

Additionally, all patients and healthy controls will be invited to voluntarily sign the informed consent form.

### 2.4. Diagnostic Criteria

The diagnostic criteria for ischemic stroke (cerebral infarction) are as follows [38]: (1) most acute onset occurs in the static state, as dynamic onset patients are more common in cardiogenic cerebral infarction, and some cases may have transient cerebral ischemia attacks before onset; (2) aggravation or fluctuation is present; (3) clinical manifestations depend on the size and location of the infarct focus, mainly the symptoms and signs of focal neurological deficits, such as hemiplegia, paresthesia, aphasia, and ataxia, with some patients having headaches and vomiting, in coma, and others with whole-brain symptoms; and (4) there is confirmation via brain CT or MRI.

#### 2.4.1. Inclusion Criteria

The inclusion criteria for patients with stroke are as follows: (1) diagnosis of ischemic stroke confirmed by brain CT or MRI; (2) left cerebral ischemia (including the stroke patients with anterior and posterior circulation infarction) with right limb hemiplegia; (3) first-ever clinical stroke occurring between 2 weeks and six months; (4) aged between 30 and 70 years; (5) stable medical condition, Brunnstrom stages I–V; and (6) written informed consent to participate.

The inclusion criteria for healthy controls include the following: (1) can be matched to the patient's age, gender, and education; (2) has no acupuncture treatment in the last month; (3) has no history of head trauma, psychiatric disease, neurological disease, or physical activity disorder; (4) has no MRI contradictions; and (5) has provided written informed consent.

#### 2.4.2. Exclusion Criteria

The exclusion criteria are as follows: (1) a history of previous traumatic brain injury, tumor, or epilepsy; (2) bilateral hemiplegia; (3) critical organ failure, such as heart, lung, liver, and kidney failure; (4) severe cognitive impairment and inability to cooperate; (5) claustrophobia, wearing a pacemaker, intracranial metal implants, or skull defects; (6) severe cervical lesions including severe cervical spinal stenosis and cervical instability; (7) women who are pregnant or lactating; (8) contradictions for MRI scans; (9) currently participation in another clinical trial that would affect the evaluation results of this study; or (10) fear of needling, needle phobia, and fainting with needles.

#### 2.4.3. Dropout Criteria

During the trial period, participants meeting the following criteria will be excluded from the study: (1) occurrence of serious adverse events; (2) missing more than 3 of 10 acupuncture treatment sessions; (3) important violations in the protocol implementation; or (4) withdrawal from the study in light of the participants being unwilling to continue.

### 2.5. Randomization and Allocation

After meeting the selection criteria, 40 eligible patients will be randomly assigned into either the experimental group or the control group at an allocation ratio of 1 : 1 after signing written informed consent forms. The random sequence will be created by a third-party professional statistician of Shenzhen University using STATA 12.0 software (StataCorp, Texas, USA). The randomization list will only be seen by this research coordinator and will be concealed from other study personnel. Participant assignments will be hidden using sequentially numbered, opaque, sealed envelopes. The research coordinator that assigns the groups will not participate in the patients' inclusion, the subsequent trial process, or the assessment of results.

### 2.6. Blinding

The trial will be conducted using a patient-assessor blind design. The patients, assessors, and statisticians will not be aware of which group the patients are in. They will only be told whether data will belong to group A or group B, but they will not be aware of which group is the experimental group or the control group. Furthermore, they will not even know whether the trial is designed to be a noninferiority trial, an equivalent trial, or a superiority trial, thus avoiding human factors affecting the results of the data analysis.

Regarding acupuncture implementation, acupuncture doctors cannot be blinded, so unblinded acupuncture doctors will apply the acupuncture intervention depending on the patients' intervention arm.

The blind method will be applied throughout the whole study process. A member of the trial staff will supervise the process of blind implementation and will disclose the blind code after the statistical analysis is completed. Unblinding will be considered only in a critical medical emergency.

### 2.7. Treatment

#### 2.7.1. Basic Drug Treatment

Combined with their past stroke-related risk factors and current medical histories, all patients will receive secondary prophylactic drug treatment for stroke, including antihypertensive, hypoglycemic, antiplatelet, and hypolipidemic drugs.

#### 2.7.2. Conventional Rehabilitative Interventions

Conventional rehabilitation treatment will be administered 5 days per week, 10 sessions (2 consecutive weeks):

- Physical therapy [5]: PT will include active and passive activity of affected limb joint, neurodevelopmental therapy, balance function training, antispasmodic treatment, muscle strength training, and walking gait training.
- Occupational therapy [5]: OT will include therapeutic activities for the upper limbs and hands of the affected side, activities of daily living, and training for the use of auxiliary equipment.

PT and OT will be performed by professional physiotherapists with at least 5 years of clinical experience. Each patient's pre- and postrehabilitative intervention will be conducted by the same physiotherapist.

#### 2.7.3. Experimental Group

The experimental group will receive acupuncture at temporal three-needle 5 times per week for 2 weeks following basic drug treatment and conventional rehabilitative treatment. The details of temporal three-needle therapy are presented in Table 2; the therapy will comply with the Standards for Reporting Interventions in Clinical Trials of Acupuncture (STRICTA) checklist [39].

**Figure 2:**
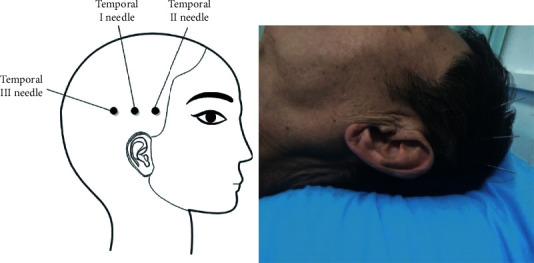
Locations of temporal three-needle therapy in the experimental group (the use of the picture has been allowed by the author Tongyan Liu and the patient).

The temporal I needle will be placed on the hairline 2 cun above the apex of the ear; the temporal II needle and temporal III needle will be placed 1 cun from the temporal I needle on either side in the horizontal direction [13] (Figure 2; the use of the picture has been allowed by the author Tongyan Liu and the patient). After routine disinfection of the scalp, disposable stainless-steel needles (0.30 × 25 mm) (Huanqiu, Suzhou Acupuncture & Moxibustion Appliance Co., Ltd., Jiangsu Province, China) will be manually inserted at each acupoint. After the acupuncturists insert needles into the acupoints at a certain depth and twist them for a while, patients report conscious sensations, such as soreness and numbness, in the acupuncture location, which indicates needle sensation “De Qi.” To treat motor dysfunction, the needles are rotated at 100 revolutions or more per minute for 1 minute at every 5 minutes over 30 minutes.

#### 2.7.4. Control Group

The control group will receive acupuncture at the MS6 from Qianshencong (EX-HN1) to Xuanli (GB6) [20] following basic drug treatments and conventional rehabilitative treatments. The details of acupuncture at MS6 are presented in Table 2; this treatment will comply with the STRICTA checklist [39] as well.

### 2.8. Outcome Assessment

The outcome measurements include functional connectivity (FC), regional homogeneity (ReHo), amplitude of low-frequency fluctuations (ALFF), the Fugl–Meyer assessment of upper limb (FMA-UL), the modified Barthel Index (MBI), and adverse events. FC, ReHo, and ALFF will be measured by rs-fMRI. The FMA-UL, MBI, and adverse events (AEs) will be assessed by three experienced rehabilitative assessors.

Additionally, the study duration will be 2 weeks. Outcomes will be assessed at baseline and after 2 weeks of intervention. Each subject will have the same assessor for their pre- and posttreatment evaluations.

#### 2.8.1. Primary Outcome

The primary outcome is resting-state FC. Functional brain alterations in this study will be more sensitive than structural MRI and behavioral performances in detecting the neuromodulatory effect of temporal three-needle therapy.

Resting-state FC analyses can assess synchronous activity between brain regions and be used to study the brain in healthy and disease groups on the brain-network level, which will help to understand (a) markers for diseases, (b) paths to investigate disease mechanisms, and (c) predictors of clinical course [40]. Resting-state FC could serve as a biomarker of motor function recovery in stroke patients with hemiplegia [41]. Stronger FC after stroke correlates with better behavioral outcome [42]. Resting-state FC analysis may offer an invaluable approach to explore functional network reorganization in the brain after stroke. In this study, brain FC plasticity within and beyond the sensorimotor network will be measured by rs-fMRI.

#### 2.8.2. Secondary Outcomes

The secondary outcomes including ReHo, ALFF, FMA-UL, and MBI will be measured at the same time points of the primary outcome. Brain regional activity (ReHo and ALFF) will be assessed via rs-fMRI. FMA-UL, MBI, and AEs will be assessed by the abovementioned three experience rehabilitative assessors.

ReHo can be used to explore the consistency of neuronal activity in regional brain areas at resting state, which may be a potential biomarker of the neural substrates associated with hand function following stroke [43] and provide an effective tool for evaluating the efficiency of rehabilitative therapies after stroke [44]. An increase in ReHo value indicates that the consistency of neuron activity tends to be increased.

ALFF can reflect the intensity of the nerve activity of a single voxel and hemodynamic BOLD information on neural activity [45]. In addition to FC, regional ALFF may provide complementary information on the neural mechanism [46]. After brain injury, the blood flow of the brain decreases, and the local ALFF value shows a downward trend.

The FMA-UL is used to only assess motor function of the upper limb in stroke patients. Because the motor function of the upper limb is the most difficult to recover after stroke, we focused on upper limb motor dysfunction in this study. The FMA-UL has been shown to be useful for measuring arm and hand motor function, and 30-item assessment shows a longitudinally stable item difficulty order and is valid for measuring volitional arm motor ability over time [47]. The FMA has a total of 100 points for normal motor function. The maximum score for UL is 66. The evaluation includes measuring reflex activity, flexor synergy, extensor synergy, and movement combining synergies, movement out of synergy, elbow, shoulder, wrist, and hand function, and relates coordination and speed. An ordinal scale applied to each item is used: 0 = cannot be performed, 1 = can be partially performed, and 2 = can be completely performed.

The MBI has shown good properties for measuring disability in stroke patients [48] and is the most prevalent functional outcome scale in stroke trials. The MBI covers 10 domains: feeding, grooming, dressing, bathing, toilet transfers, bowel and bladder continence, ambulation, moving between wheel chair bed and back to the wheel chair, and stair climbing. Each item has five levels, with level 5 representing the greatest independence.

### 2.9. Brain MRI Protocol

Healthy controls will undergo a baseline brain MRI scan, and patients in the experimental group and the control group will undergo brain MRI scans at baseline and after treatment. All brain MRI scans will be performed with a 3.0 Tesla scanner (SIEMENS Skyra, Germany) in the Department of Radiology, Shenzhen Nanshan District People's Hospital. The parameters of the sequences were as follows.

Structure MRI: prior to brain functional imaging scan, T1-weighted images of three-dimensional whole-brain structure in sagittal position will be collected using MPRAGE sequence with the following parameters: repetition time (TR)/echo time (TE) = 2530/2.01 ms, flip angle (FA) = 7°, field of view (FOV) = 256 × 256 mm^2^, matrix = 256 × 256, 176 contiguous axial slices, slice spacing = 0.0 mm, and slice thickness = 1.0 mm.

T2 FLAIR (fluid-attenuated inversion recovery):T2 FLAIR will be collected using a turbo spin echo (TSE) sequence with the following parameters: TR = 8500 ms, TE = 102 ms, and flip angle = 160°.

Rs-fMRI: resting-state functional images will be performed using an echo planar imaging (EPI) sequence with the following parameters: TR/TE = 2000/30 ms, FA = 90°, number of slices = 36, matrix = 64 × 64, FOV = 224 × 224 mm^2^, and slice thickness = 3.5 mm.

### 2.10. Data Collection, Management, and Monitoring

The design, modification, and final confirmation of the case report form (CRF) will be jointly conducted by Shenzhen Nanshan District People's Hospital and researchers. The CRF must ultimately be approved by Shenzhen Nanshan District People's Hospital. Electronic CRF will be utilized for data collection. The data entry will be performed separately by two independent staff.

Specialized data manager will be used to maintain the integrity, validity, and correctness of the data and to fully check the primary and secondary outcomes and security indicators specified in the plan to ensure the accuracy and completeness of these data.

Shenzhen Nanshan District People's Hospital is responsible for data collection, data entry, data verification and query, medical coding, blind audit, data export and transmission, archiving of data, and data management.

### 2.11. Statistical Analysis

#### 2.11.1. MRI Data Analysis

The DPABI toolkit [49] will be used to analyze the image data on MATLAB2016 (MathWorks) to detect any functional changes (FC, ReHo, and ALFF) in brain neuroplasticity due to temporal three-needle treatment. After data preprocessing, data-driven approach will be conducted to explore neuroplastic changes in the two patient groups and the healthy control group. Data-driven approaches can automatically obtain the brain functional network from the data [50]. Independent component analysis (ICA) based on data-driven approaches is not required to assume a priori that the MRI signal can be separated directly into a series of maximally independent components and associated time courses [51, 52] and different components corresponding to the corresponding resting-state network. FDR correction will be used for multiple comparisons to control the false positivity rate. Pearson's correlation analysis will be utilized to study the relationship between the rs-fMRI metrics and behavioral data.

#### 2.11.2. Clinical Behavioral Data Analysis

In this study, statistical analyses will be performed by intent-to-treat (ITT) analysis and per-protocol (PP) analysis. The ITT analysis will include not only all patients with valid results but also randomized patients who were discontinued for a period of time for some reason or transferred to another group. For missing values, we will carry over the last observation results to the endpoint so that the number of patients at the endpoint of each group will be the same as that at the beginning of the trial. The PP analysis will be applied to subjects who adhere to the protocol, have good compliance, and complete the process. When differences in the results exist between the two analyses, the final clinical trial results will be discussed and explained. In this study, only those who received more than 70% of the interventions will be considered satisfied compliance. That is to say, in total number of the 10 treatments, subject missing more than 3 treatments will be excluded from the PP analysis due to violation of the protocol.

All differences in the baseline demographic and clinical characteristics between the two patient groups will be analyzed with the chi-squared test or Fisher's exact test for categorical data and with the *t*-test or Wilcoxon rank-sum test for continuous values. Continuous variables will be shown as the means, SDs. Categorical variables will be described as frequencies or percentages. 

All tests will be two-sided, and a *p* value <0.05 will be considered statistically significant. If the difference of the primary endpoint between the experimental group and the control group was statistically significant, the results of the experimental group are superior to that of the control group, and the intervention method could be considered as effective. Therefore, we firstly set the type I error of the primary endpoint at 0.05. If significant, we then go to test the secondary endpoint at 0.05 type I error.

The changes in the FMA and the MBI between pre- and postacupuncture therapy will be analyzed using repeated measures analysis of variance (ANOVA), This analysis will be used to compare differences and to examine the group effect, time effect, and time-group interaction effect.

A post hoc test will be conducted. Within-group differences between pre- and posttreatment will be compared using the paired *t*-test or Wilcoxon signed rank test for continuous data. 

AEs will be tabulated and summarized using descriptive statistics. The AEs incidence of each group will be confirmed as evaluation index, and the computation formula is as follows: AEs incidence% = (number of AEs/total number of cases in this group) × 100%. The current version of STATA 12.0 statistics (StataCorp, Texas, USA) will be used for statistical analyses.

### 2.12. Quality Control

Shenzhen Nanshan District People's Hospital will monitor the following aspects of the trial regularly and strictly: the division and training for the research staff, file management, informed consent, protocol compliance, subject recruitment, filling in the CRF, intervention, quality assured system, statistical analysis, and data management.

### 2.13. Investigation of Adverse Events and Safety

Intervention-related AEs might include edema due to bleeding, bending and breaking of the needle, fainting during acupuncture, and retained needle after treatment. During the course of the study, any AEs including the onset of clinical symptoms, degree of symptom severity, persistence time, treatment, and sequelae will be recorded. AEs will be compared between patients in the experimental group and the control group and will be recorded in the CRF and assessed by the research staff at each visit. Serious AEs will be reported to the ethics committee immediately.

### 2.14. Trial Status

Currently, this trial is at the recruitment phase with some subjects being involved.

## 3. Discussion

The similarities between the temporal three-needle and MS6 are as follows: (1) the two are both locating on the side of the skull and (2) the temporal three-needle and MS6 can treat contralateral central motor dysfunction [11, 20]. The differences between the temporal three-needle and MS6 are as follows: (1) the acupoint projecting brain regions are different: the temporal three-needle projects the sensory region and the motor region including the precentral gyrus and the postcentral gyrus, which may be responsible for sensory function and motor function of the limbs [13], and MS6 projects the precentral gyrus [21], which manages autonomous limb movement [20]; (2) the acupoint-related meridians are different: the temporal three-needle connects the Gallbladder Meridian of Foot-Shaoyang (GB), Bladder Meridian of Foot-Taiyang (BL), Stomach Meridian of Foot-Yangming (ST), and Sanjiao Meridian of Hand-Shaoyang (SJ); MS6 connects the Du meridian (DU), BL, and GB; (3) the acupoint stimulation sensation is different: the temporal three-needle is located at the temporal sutures which have a high degree overlap, and the gaps left by this congenital development process can help the needle sensation of the extracranial scalp penetrate deeper into the skull [10, 11, 13]; however, the MS6 stimulation location includes the parietal bone, frontal bone, and temporal bone, which are not the thinnest part of the skull, so the transmission of the acupuncture sensation may be weaker than that of the temporal three-needle. In summary, because of its different projected cortex, diverse-related meridian system, deeper stimulation sensation, and wider dysfunctional treatment (the temporal three-needle focuses on both motor dysfunction and sensory dysfunction), temporal three-needle therapy is now more widely used for ischemic stroke in China.

Studies have found that abnormal brain structures and functions exist in patients after stroke compared with age- and gender-matched healthy controls [53, 54]. In this trial, we will aim to investigate the neuromodulatory effect of sensorimotor network FC of temporal three-needle therapy for ischemic stroke patients with motor dysfunction. First, we will compare the differences in baseline rs-fMRI metrics between the healthy subjects and the stroke patients to confirm the difference between the healthy subjects and stroke patients. Second, to determine the difference due to the temporal three-needle treatment, we will compare the changes in the rs-fMRI metrics between the experimental group and the control group to demonstrate whether motor function alteration is related to brain functional neuroplastic changes after temporal three-needle treatment. Although a large number of studies on the use of temporal three-needle therapy for the treatment of motor dysfunction, speech disorders, dysphagia, and depression after stroke have indicated clinical behavioral effects [11, 14–18], to the best of our knowledge, our study will be the first randomized neuroimaging trial with both a positive control group and a healthy control group to examine the actual effects by means of rs-fMRI.

This study has several strengths. First, the implementation of rigorous randomized, patient-assessor blind, controlled, neuroimaging trial will provide visual and objective evidence about how temporal three-needle treatment improves motor dysfunction in ischemic stroke patients. Second, the combined assessments of rs-fMRI and behavioral data will be used to identify the neuromodulatory effects of temporal three-needle therapy in stroke patients. Third, research based on the FC within the sensorimotor network, and between the sensorimotor network and nonmotor network, is better for understanding the mechanism of temporal three-needle therapy in stroke according to the “scalp-cortex corresponding theory.”

Some limitations of this study should be noted. First, the major limitation of this protocol is its lack of long-term follow-up assessments. Second, there is no sham acupuncture or blank control comparison group for observation. Third, this trial will be conducted in a single center. Fourth, only the patients of left cerebral ischemia with right limb hemiplegia will be recruited in this protocol, and the patients of right cerebral ischemia with left limb hemiplegia will not be temporarily considered enrolled in this trial. We plan to resolve the aforementioned limitations in the future.

In conclusion, the results of this study are expected to explore the neuromodulatory effects and characterize the central mechanism of temporal three-needle treatment on motor dysfunction in hemiplegic stroke patients from the network-level viewpoint of sensorimotor network functional plasticity and to promote widespread application in real-world practice.

## Figures and Tables

**Table 1:** Study schedule for enrollment, interventions, and assessments.

Study period				
Items	Baseline		Treatment phase	Outcomes assessment
Time points	−1 to −3 days	0 weeks	1–2 weeks	3 weeks
*Enrollment*				
Eligibility screen	×			
Informed consent	×			
Examination		×		
Randomization		×		
*Intervention*				
Healthy control group (*n* = 20)				
Experimental group (*n* = 20)			×	
Control group (*n* = 20)			×	
*MRI scan*				
Healthy control group		×		
Experimental group		×		×
Control group		×		×
*Assessment (only in the experimental group and the control group)*				
FMA		×		×
MBI		×		×
*Safety*				
Adverse events			×	×

**Table 2:** STRICTA checklist (details of intervention).

	Items	Details
(1) Acupuncture rationale	(1a) Style of acupuncture	Temporal three-needle therapy based on the rationale system of the meridian acupoint theory in Chinese traditional medicine, modern neurological theory, and anatomical rationale [10, 11]
	(1b) Reason for the treatment provided, based on the historical context, literature sources, and/or consensus methods, with references where appropriate	Acupoints to be used in this study were temporal three-needle located at the projection area of the sensory area and the motor area on the temporal ear [11]. Based on many previous studies, temporal three-needle therapy is widely used for stroke treatment in China [11, 14–18]
	(1c) Extent to which treatment was varied	Only the experimental group will receive temporal three-needle therapy

(2) Details of needling	(2a) Number of needle insertions per subject per session	3
	(2b) Names of points used	Temporal three-needle
	(2c) Depth of insertion, based on a specified unit of measurement	0.5 inches deep
	(2d) Response sought	“De Qi” sensation
	(2e) Needle stimulation	Manual operation
	(2f) Needle retention time	30 minutes
	(2g) Needle type	Sterilized stainless-steel needle measuring 0.30 × 25 mm (Huanqiu, Suzhou Acupuncture & Moxibustion Appliance Co., Ltd, Jiangsu Province, China)

(3) Treatment regimen	(3a) Numbers of treatment sessions	10 sessions (2 consecutive weeks)
	(3b) Frequency and duration of treatment sessions	30 minutes per day, 5 days per week

(4) Other components of treatment	(4a) Details of other interventions administered to the acupuncture group	All patients will receive basic drugs treatment and conventional rehabilitative treatments (including PT and OT)
	(4b) Setting and context of treatment, including instructions to practitioners and information and explanations to patients	The study will be conducted in Shenzhen Nanshan district People's Hospital, and all information will be provided to the patients

(5) Practitioner background	(5) Description of participating acupuncturists	Acupuncturists after completing 5 years of Chinese medicine undergraduate course with more than 10 years of clinical experience will perform the temporal three-needle interventions

(6) Control or comparator interventions	(6a) Rationale for the control or comparator in the context of the research question, with sources that justify this choice	MS6 will be selected as the comparator in the control group based on traditional Chinese medicine theory
	(6b) Precise description of the control or comparator; if sham acupuncture or any other types of acupuncture-like control is used, provide details as for items 1 to 3 above	Based on previous studies [19, 22], MS6 will be mainly selected as the comparator for temporal three-needle therapy in motor dysfunction with stroke patients; in addition to the basic drug treatments and conventional rehabilitative treatment, only the control group will receive MS6 therapy; item 2 (details of needling) and item 3 (treatment regimen) are the same as the experimental group

## Data Availability

Data and materials are available upon reasonable request from the co-first authors.

## Abbreviations

MRI: Magnetic resonance imaging
fMRI: Functional magnetic resonance imaging
Rs-fMRI: Resting-state functional magnetic resonance imaging
FC: Functional connectivity
ReHo: Regional homogeneity
ALFF: Amplitude of low-frequency fluctuations
FMA: Fugl-Meyer assessment
MBI: Modified Barthel index
CRF: Case report form.

## Additional Points

Recruitment began in March 2018 (Protocol version 1.0).

## Supplementary Materials

Supplementary Materials SPIRIT2013 checklist: recommended items to address in a clinical trial protocol and related documents.
